# MONTAGE: a new tool for high-throughput detection of mosaic copy number variation

**DOI:** 10.1186/s12864-021-07395-7

**Published:** 2021-02-24

**Authors:** Joseph T. Glessner, Xiao Chang, Yichuan Liu, Jin Li, Munir Khan, Zhi Wei, Patrick M. A. Sleiman, Hakon Hakonarson

**Affiliations:** 1 Department of Pediatrics, Children’s Hospital of Philadelphia, 3401 Civic Center Blvd, Philadelphia, PA 19104 USA; 2 Department of Pediatrics, Perelman School of Medicine, University of Pennsylvania, 3400 Civic Center Blvd, Philadelphia, PA 19104 USA; 3 New Jersey Institute of Technology, Newark, NJ 07102 USA

**Keywords:** Mosaicism, Mosaic, Copy number variation, Genomics

## Abstract

**Background:**

Not all cells in a given individual are identical in their genomic makeup. Mosaicism describes such a phenomenon where a mixture of genotypic states in certain genomic segments exists within the same individual. Mosaicism is a prevalent and impactful class of non-integer state copy number variation (CNV). Mosaicism implies that certain cell types or subset of cells contain a CNV in a segment of the genome while other cells in the same individual do not. Several studies have investigated the impact of mosaicism in single patients or small cohorts but no comprehensive scan of mosaic CNVs has been undertaken to accurately detect such variants and interpret their impact on human health and disease.

**Results:**

We developed a tool called Montage to improve the accuracy of detection of mosaic copy number variants in a high throughput fashion. Montage directly interfaces with ParseCNV2 algorithm to establish disease phenotype genome-wide association and determine which genomic ranges had more or less than expected frequency of mosaic events. We screened for mosaic events in over 350,000 samples using 1% allele frequency as the detection limit. Additionally, we uncovered disease associations of multiple phenotypes with mosaic CNVs at several genomic loci. We additionally investigated the allele imbalance observations genome-wide to define non-diploid and non-integer copy number states.

**Conclusions:**

Our novel algorithm presents an efficient tool with fast computational runtime and high levels of accuracy of mosaic CNV detection. A curated mosaic CNV callset of 3716 events in 2269 samples is presented with comparability to previous reports and disease phenotype associations. The new algorithm can be freely accessed via: https://github.com/CAG-CNV/MONTAGE.

**Supplementary Information:**

The online version contains supplementary material available at 10.1186/s12864-021-07395-7.

## Background

Mosaicism is non-integer CNV resulting from a mixture of deleted and diploid or duplicated and diploid cells. Mosaic CNV creation mechanisms include: chromosome nondisjunction, anaphase lag, and endoreplication. Mosaicism was first studied in fruit flies by Alfred Sturtevant and Curt Stern demonstrating mitotic recombination. “Somatic mosaicism” terminology was used by C. W. Cotterman in his seminal paper about antigenic variation [1]. Mosaic CNV detection is important in clinical settings for accurate assessment and estimate of disease recurrence risk [2–5].

Almost all CNV detection algorithms interpret a splitting of genotypes into allelic imbalance to mean duplication. However, splitting of genotypes into allelic imbalance clusters means duplication only when paired with a gain in intensity, otherwise the event is actually a mosaic deletion. In this way, mosaicism is often incorrectly classified or missed entirely by conventional CNV detection algorithms.

Mosaicism calls from publically accessible programs, such as R-GADA-MAD, BAFSegmentation, Mocha, and triPOD have provided insight into the prevalence and impact of mosaic CNVs [6–8]. However, we were motivated to create a computationally efficient and easy to perform algorithm, particularly at the scale needed for large projects. The goal of the algorithm was to deliver results in a high-throughput way with few spurious calls and to successfully detect mosaic events multiple megabases in size. Previous studies have been limited by small sample size to achieve accurate frequency estimates ([9, 10], Bonnefond et al. 2013, Schick et al. 2013, [11, 12], and Rodriguez-Santiago et al. 2010).

Mosaic Alteration Detection (MAD) is among the most commonly used computational tool to identify mosaic events using both B-allele frequency (BAF) and log R ratio (LRR) values from SNP array data. The MAD method first performs a segmentation procedure using the GADA algorithm (Pique-Regi R, Caceres A, Gonzalez JR. BMC Bioinformatics, 2010), and then searches for aberrant segments (mosaic regions) with B-deviations (Bdev) different from zero. Bdev occurs in mosaic regions when the locus has a mixture of genotypes from normal and mosaic tissue. The detected mosaic regions are further classified as copy-loss, copy-gain, or copy-neutral events based on the alteration of the LRR from baseline. The MAD method is implemented as a package in R. and the program is available at GitHub (https://github.com/isglobal-brge/MAD). The state of mosaic alterations is determined based on log2-ratio segment values together with the percentage of normal heterozygous (BAF ∼ 0.5) and homozygous (BAF ∼ 0 or 1) probes. While MAD has been successfully applied in several mosaicism projects (Forsberg et al. 2012, 2014 [11];), the program installation remains difficult requiring many undocumented dependencies including the R package devtools rendering it difficult to operate.

It’s important to be aware that inherited alleles at some loci may appear to affect the probability of somatic mutations, and at other loci they may constitute objects of positive or negative clonal selection. Several specific mosaic CNVs are strongly associated with future risk of hematological malignancies [8]. Loh et al. evaluated blood-derived DNA from 151,202 UK Biobank participants genotyped with Affymetrix arrays, finding 8342 mosaic CNVs ranging 50 kb–249 Mb by using phase-based programming (false discovery rate ≈7.5%). Mosaic deletions were observed more frequently in males while mosaic duplications were observed more frequently in elderly and male samples and copy number neutral loss of heterozygosity (CNN-LOH) affected the sexes equally.

In addition to the large number of samples from the Center for Applied Genomics at Children’s Hospital of Philadelphia biobank presented here (Supplementary Table 1), we also explored the pediatric and adolescent age range (Supplementary Fig. 1), providing further insights into early mosaic detection possibilities. Taken together, our results reveal clonal expansions with a wide range of effects on human health.

### Implementation

Due to current limitations and difficulties in available mosaic genomic events callers, we have developed a mosaic calling tool programmed in Perl and freely available through our GitHub webpage https://github.com/CAG-CNV/MONTAGE. We wrote the Perl code to be flexible in terms of column order and column inclusion in sample based input files. The minimum column requirement for input files is SNP Name, BAF, and LRR. SNP Name with associated chromosome and base pair position can be specified separately or combined in the input. Sorting by chromosome and position (if not done already) is the first step. This is the most run time consuming step at 25 s. If sorting is detected to be done already, only the mosaic CNV detection portion of the code runs taking 10 s per sample (Table 1).

**Table 1:** Performance Comparison of Mosaic CNV Detection Tools

Algorithm	Install	Runtime	Sensitivity	Specificity	URL
MONTAGE	Easy	Short (35 s/10s^a^)	Good(1/1)	Good(0/0)	https://github.com/CAG-CNV/MONTAGE
MoChA	Difficult	Long (1m1s^b^)	Good(1/1)	Good(0/0)	https://github.com/freeseek/mocha
RGADA-MAD	Difficult	Short (14 s)	Low(0/1)	Low(1/0)	https://github.com/isglobal-brge/MAD
BAFSegmentation	Easy	Long (1m14s)	Good(1/1)	Low(186/0)	http://baseplugins.thep.lu.se/wiki/se.lu.onk. BAFsegmentation
triPOD	Easy	Very Long (10 m)	Low(0/1)	Low(0/0)	https://github.com/jdbaugher/tripod

Install ease based on actual setup with non-superuser credentials, not exclusively the documented setup instructions provided by the algorithm. Runtime listed per sample 610 k density SNP microarray. ^a^Sorted by chromosome and position input file. ^**b**^Eagle phasing pipeline (1 m) and Chromosomal alterations pipeline (1 s) steps included. Sensitivity and Specificity based on running the same sample data through each algorithm and comparing results. In parenthesis is Observed / Expected mosaic CNV calls. See Fig. 7 for additional Sensitivity/Specificity analysis where we demonstrate in 755 samples a 0.975 false positive rate 0.344 (MONTAGE) vs. sensitivity of 0.920 at false positive rate 0.598 (MoCha) vs. sensitivity of 0.280 at false positive rate 0.627 (RGADA-MAD)

Since Windows computers are needed to run GenomeStudio to load idats and export BAF/LRR signal files, we remove any Windows carriage returns. We remove SNPs that failed to UCSC Liftover or low call rate SNPs marked with Position REMOVE. If not sorted, sort by chromosome and position (for linear runtime). Remove position 0 SNPs. A bash awk statement embedded in the Perl code efficiently performs a sliding window of 1 MB with 1 MB increments to roughly assess potential regions of mosaicism. The algorithm monitors position modulus window, if diff< 0 then report window: CHIP REGION AB ABLow ABHigh AAorBB AvgLRR BAF_SD(0.1–0.9). To define deletion, we take q3 + 1.5 interquartile range (iqr) as BAF standard deviation (SD) threshold and q1–1.5 iqr as LRR average threshold. To define duplication, we take q3 + 1.5 iqr as BAF SD threshold and q3 + 1.5 iqr as LRR average threshold. PennCNV script clean_cnv merges fragmented mosaic CNVs in neighboring genomic intervals. We record the first and last base pair position of mosaic evidence in these intervals to provide specific breakpoints (Fig. 2).

Methods used include programming in Bash, Perl, and R.

Efficient and minimal dependency coding allows for rapid ease of deployment of the software.

The BAF ranges used are tallied in the following intervals: (0–0.1) (0.1–0.4) (0.4–0.6) (0.6–0.9) (0.9–1). We use average LRR for each 1 Mb window in comparison with genome-wide average LRR. This presents the key command in the script. We note that our current version of MONTAGE, runs at 35 s per sample in a sample independent manner vs. 10 s if the input BAF/LRR signal file is sorted by chromosome and position.

#### Data preprocessing

We dynamically assess the column header to determine the presence and order of SNP Name, Chromosome, Position, B Allele Freq, and Log R Ratio column data in the user provided input files. These inputs may be generated by exporting text files from graphical user interface on Windows: Illumina GenomeStudio or Affymetrix Genotyping Workbench. Alternatively, these files can be generated by exporting text files from command line on Linux: Illumina iaap or Affymetrix apt. The flexibility to include only SNP Name, B Allele Freq, and Log R Ratio column data is allowed to minimize the disk space footprint of input data files, provided a separate map file linking SNP Name to chromosome and position. SNP microarray, whole exome sequencing and whole genome sequencing data are all supported input data types based on normalization of read depth to generate LRR signal and clustering of allele depth to generate BAF.

#### Mosaicism detection

Using an ultra-efficient awk bash command, we are able to run an optimal non-overlapping sliding window algorithm to determine BAF in the mosaic deletion indicative ranges of (0.1–0.4) and (0.6–0.9) as well as the standard deviation of these observations to determine clarity (lack of noise) in the signal observed in a given sample. We implemented a sliding window approach to assess these BAF intervals for allelic imbalances and strong deviations from expected values. Average LRR values across the sliding window interval classify the mosaic CNV as a deletion or duplication relative to normal diploid copy number. High standard deviation of BAF (0.1,0.9) regions were prioritized (for those samples passing quality control with acceptably low genome-wide standard deviation of BAF).

#### Mosaicism breakpoint refinement

PennCNV (version 1.0.4) component script clean_cnv was used to combine segments in close proximity into one merged mosaic CNV call. Record the first and last base pair position of mosaic evidence in these intervals to provide specific breakpoints (Fig. 2).

#### Mosaicism algorithm differences

We do not perform phasing as done in MoCha to save on computational time. We do not require or use family information as done in triPOD. We focus our code in the extensible Perl and Bash programming languages as opposed to RGADA-MAD which is written in R. We use standard modern GitHub code tracking as opposed to BAFSegmentation which is on an institutional website.

## Results

**Fig. 1:**
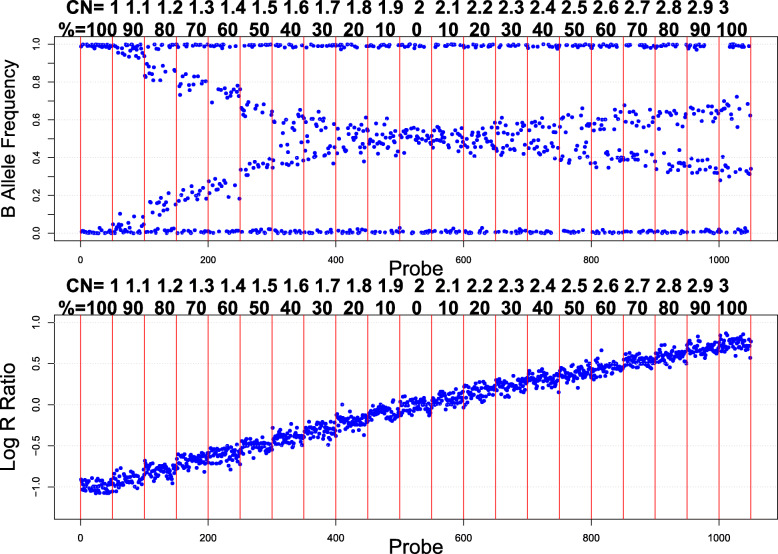
Example BAF and LRR Profiles Sampled from Various Levels of Mosaicism. Mosaic deletions have clearing of the 0.5 AB range and relatively equal banding of AAB and ABB range genotypes reflected by BAF. Less relative gain in LRR is observed in mosaic duplications, making them more difficult to detect with certainty. Data displayed is simulated to ensure consistent underlying data quality profiles for fair comparison of different mosaic copy number states

**Fig. 2:**
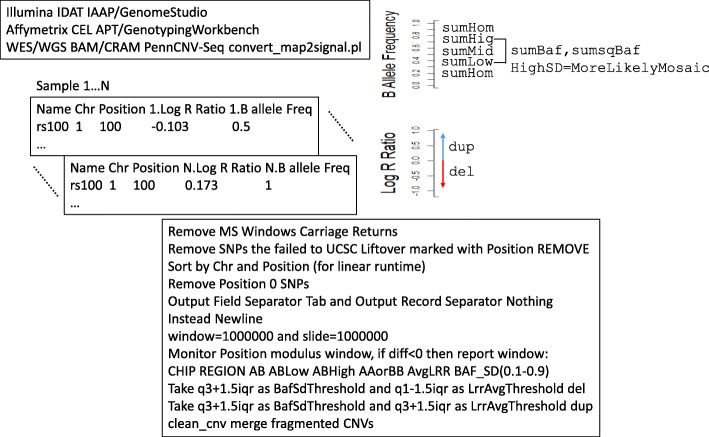
MONTAGE Algorithm Conceptual Flow Diagram. Code overview with key inputs and variables. First data is normalized to BAF and LRR values genome-wide for each sample. Then the number of deviating BAF values in a first pass sliding window are accounted to screen for potential mosaic events as an initial algorithmic step. The first quartile minus 1.5 interquartile range of the LRR paired with strong BAF deviation defines the calling threshold for mosaic deletion events. Fragmented windows meeting threshold are then merged together to form larger calls. The breakpoints are then refined based on the first and last specific base pair evidence of strongly deviating BAF within the merged window

**Fig. 3:**
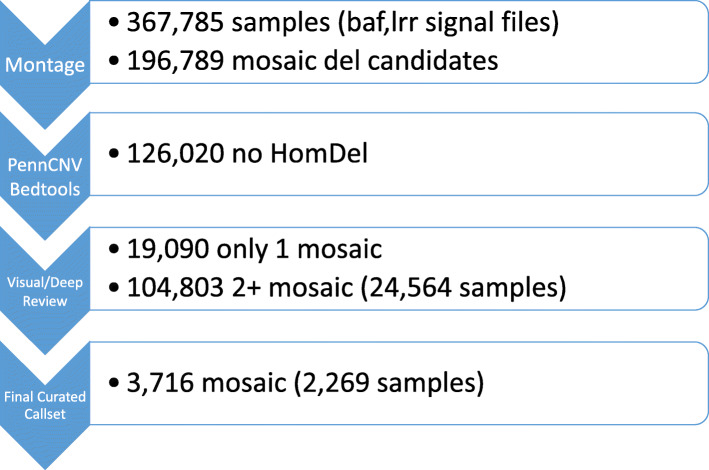
MONTAGE Filtering to Arrive at Curated Callset. Filtering of the putative mosaic CNV calls and respective size of the curated callset at each step. PennCNV calls for homozygous deletions (copy number 0) on the same samples analyzed by MONTAGE were intersected with MONTAGE initial mosaic calls using bedtools software. Further visualization of BAF/LRR underlying potential mosaic CNV calls was conducted manually by a human expert reviewer (in the case only 1 mosaic call in the sample) or by DeepCNV algorithm (in the case 2 or more mosaic calls in the sample)

We assessed the performance of existing mosaic CNV detection algorithms (Table 1). We constructed a model reference of various levels of mosaicism (Fig. 1). We measured the allelic imbalance between proper heterozygous (AB) genotypes centered on 0.5 BAF versus those outside of this region (Fig. 2 and Supplementary Fig. 2). We evaluated mosaic events in 367,785 samples and found 3716 putative mosaic events in 2269 individuals with 2/3 of the raw mosaic calls being visually validated. In total, 187,096 mosaic CNV candidates were suggested by the first pass screening of our algorithm applied to approximately 350,000 SNP-array data sets. Next, we filtered out mosaic CNV candidates with overlapping homozygous deletion calls as detected by PennCNV, since the random noise in BAF for real homozygous deletions can give a false indication of aberrant BAF banding, leaving us with 126,020 mosaic CNV candidates in 43,781 samples. The MONTAGE algorithm no longer requires PennCNV homozygous deletion calls in order to minimize runtime using an approximation subroutine. There were 51,326 mosaic CNV candidates with at least one mosaic CNV candidate > = 3 Mb in genomic span. Finally, 19,090 mosaic CNV candidates had strictly one mosaic CNV candidate > = 3 Mb, suggesting high specificity of mosaic CNV detection in these samples (Fig. 3). Therefore, we set forth visualizing the underlying BAF and LRR profiles corresponding to these mosaic CNV candidates.

**Fig. 4:**
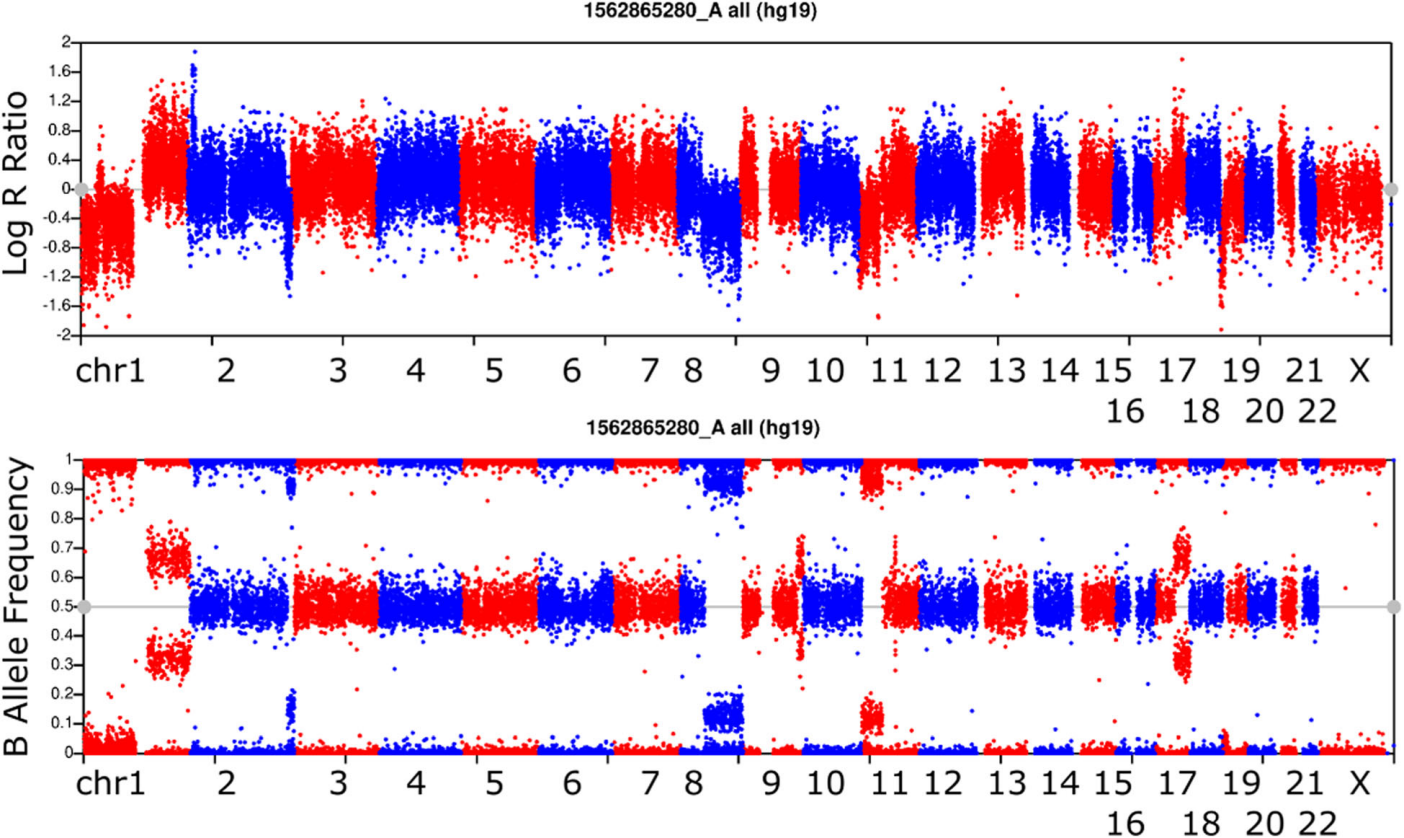
Higher Levels of Mosaicism Genome-wide LRR BAF plot representative for one Individual. Full deletion of chromosome 1p contrasted by duplication of 1q is shown along with mosaic deletions of high proportion of cells in the person’s sample on 2q, 8q, and 11p. We use alternating colors similar to a Manhattan plot for GWAS to represent the switch between chromosomes on a linear x axis

**Fig. 5:**
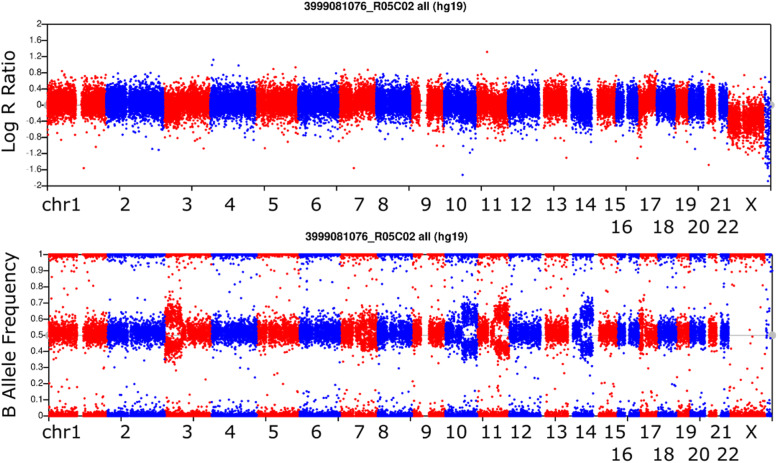
Lower Levels of Mosaicism Genome-wide LRR BAF plot representative for one Individual. Mosaic deletions of low proportion of cells in the person’s sample on 3p, 10q, 11q, and 14q

We verified ability to detect both high (Fig. 4) and low (Fig. 5) level mosaic CNV events with high sensitivity and specificity. We identified 273 putative mosaic CNV deletions in 76 out of 228 samples analyzed. Of those, 202 visually validated as true positive, confirming mosaic CNV deletions in 50 samples out of 228 samples. Median length of these CNVs was 8.5 Mb with average length of 24 Mb. The visually validated mosaic calls had at least 2 AB clearing ratio (equating to 15 AB(0.4–0.6 BAF), 16 low AB (0.1–0.4 BAF), and 16 high AB (0.6–0.9 BAF) observations in a 1 Mb genomic window. Approximately half of the mosaic calls had non-zero AB(0.4–0.6 BAF) signal indicating noise and or lower levels of mosaicism.

In order to validate the rest of the mosaic CNV candidates, we used a machine learning approach we developed called DeepCNV (in review). DeepCNV is based on a trained model of positive and negative mosaic CNV examples based on a human expert’s labeling. Using this model and images of LRR/BAF plots from PennCNV visualize_cnv which are standard and popularly used, probabilistic predictions of the mosaic CNV candidate being a true positive are output. This makes the prospect of visual validation much more tractable and reproducible with minimum generated bias.

**Fig. 6:**
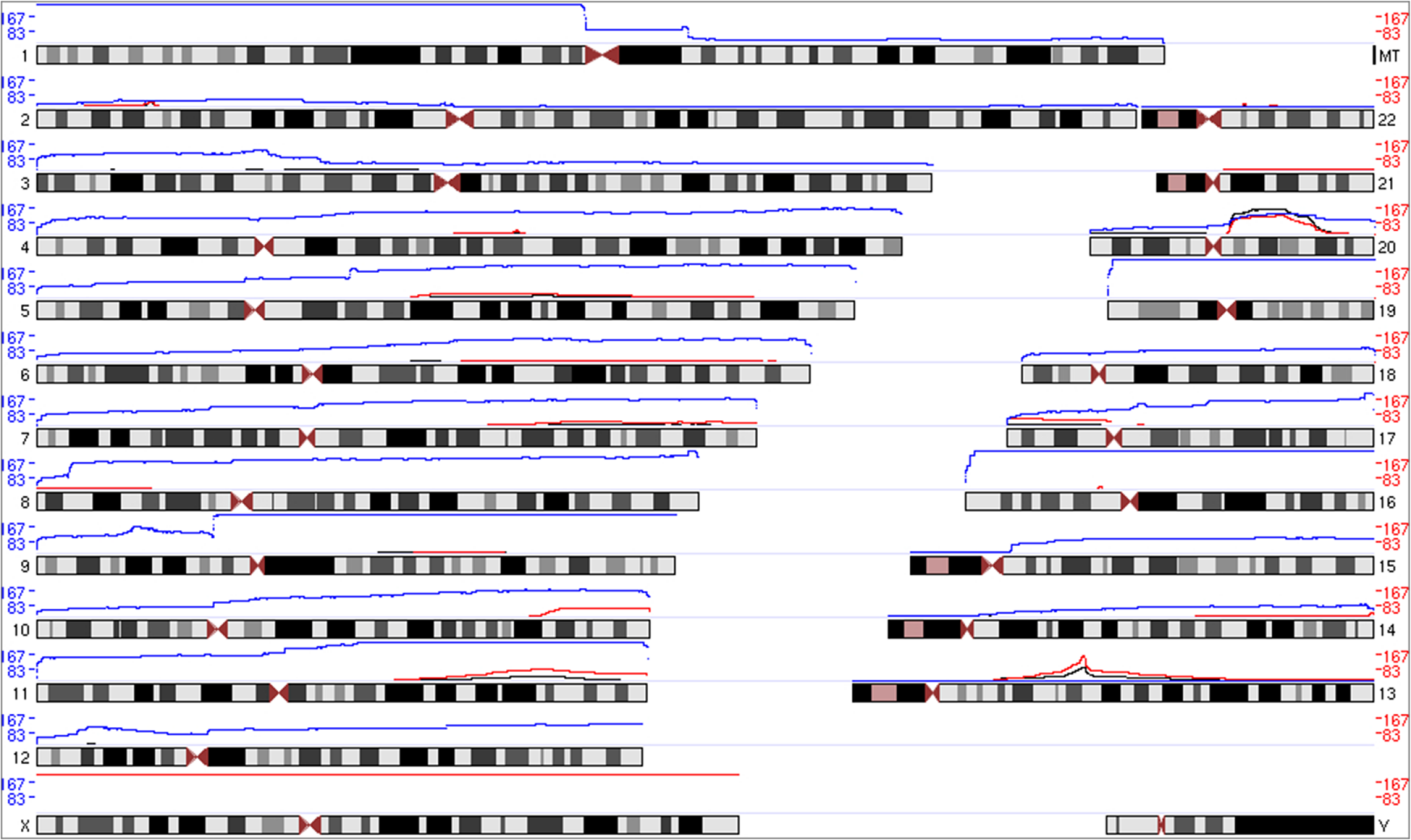
Genome-wide Count Mosaic CNVs Detected in Curated Callset in Comparison to Previous Studies. Blue line represents the current work from Montage mosaic CNV callset. Red line represents the Loh et al. mosaic CNV callset. Black line represents the other previously published mosaic CNV callsets. Previous includes: [9, 10], Bonnefond et al. 2013, Schick et al. 2013, [11, 12], and Rodriguez-Santiago et al. 2010. See Supplementary Dataset (glessner2020_hg19.txt, loh2018_hg19.txt, and prev_calls_hg19.txt)

We compared our observed mosaic CNV counts and frequencies to previous studies of mosaicism and found high concordance in genomic regions and their corresponding frequencies observed in populations (Fig. 6). In addition, when examined in the context of multiple disease phenotypes that these individuals harbored, several disease categories were associated with mosaic CNVs based on results generated using the ParseCNV software (Table 2). ParseCNV is a CNV GWAS tool [13]. Association *p*-values as low as 1E-39 were observed across phenotypes including: adhd, autism, autoimmune, cancer, congenital, healthy, and neurodegenerative [14–16]. Thirty three genomic loci were observed *p* < 5E-4 associated between mosaic events and human phenotypes. Interestingly, we observed potential protective association in healthy subjects and also may consider mosaic CNVs related to healthy subjects as negative controls [17, 18].

**Table 2:** Top 5 Significant Results *p* < 5E-4 Per Disease Category

Disease	chr	Start (Mb/hg19)	Stop (Mb/hg19)	p	OR	cases	controls	Telo/Centro	cases male	controls male
cancer	9	44	45	1.47E-39	0.065	10	251	No	4	117
cancer	9	45	46	1.81E-37	0.065	10	241	No	4	110
cancer	9	46	47	1.38E-31	0.065	10	212	No	4	97
cancer	9	43	44	1.24E-27	0.066	7	177	No	4	83
cancer	1	8	10	5.40E-24	8.701	89	19	No	65	15
autoimmune	16	1	3	5.29E-14	6.130	37	46	telomere	22	21
healthy	9	44	45	1.62E-13	3.038	73	188	No	28	93
neurodegenerative	20	41	42	1.11E-12	12.040	18	42	No	10	31
healthy	9	40	42	1.61E-12	8.797	26	22	No	14	15
neurodegenerative	20	42	45	1.45E-11	11.030	17	43	No	10	33
healthy	9	45	47	2.10E-11	2.986	62	160	No	24	78
autoimmune	19	1	3	3.77E-11	3.822	44	88	telomere	25	52
healthy	9	43	44	7.52E-11	8.797	54	130	No	20	67
healthy	15	21	23	8.05E-11	7.137	25	26	No	8	9
neurodegenerative	20	48	51	2.78E-10	12.400	14	31	No	8	21
other	9	44	46	7.82E-10	2.316	93	158	No	46	68
autoimmune	16	0	1	8.68E-10	5.724	26	34	telomere	18	16
congenital heart	22	22	23	1.11E-09	45.810	8	6	No	6	4
neurodegenerative	20	45	47	1.79E-09	10.370	14	37	No	9	29
neurodegenerative	20	31	32	4.90E-09	12.520	12	26	No	8	21
autoimmune	17	79	81	9.94E-09	7.415	19	19	telomere	9	4
congenital heart	22	20	22	2.36E-08	39.780	7	6	No	5	3
other	9	43	44	2.67E-07	2.270	68	116	No	36	51
other	9	46	47	3.99E-07	2.316	78	144	No	37	64
autoimmune	11	109	110	4.26E-07	0.000	0	113	No	72	1
congenital heart	22	19	20	8.43E-07	28.990	6	7	No	4	3
other	7	48	50	8.21E-06	7.534	14	7	No	10	5
adhd	14	107	107	1.48E-05	45.240	4	4	No	2	2
other	7	38	39	1.83E-05	6.590	14	8	No	11	5
adhd	14	106	107	2.57E-05	16.430	4	11	telomere	2	6
autism	6	20	21	3.14E-05	30.390	3	7	No	2	3
autism	6	24	26	4.27E-05	26.580	3	8	No	2	4
cancer	7	150	159	4.59E-05	4.586	18	7	telomere	10	5

Broad disease category association study of detected and curated mosaic CNV events to implicate genomic loci for disease phenotypes. The “other” disease category represents subjects without a clear primary diagnosis fitting the broad disease categories defined

**Fig. 7:**
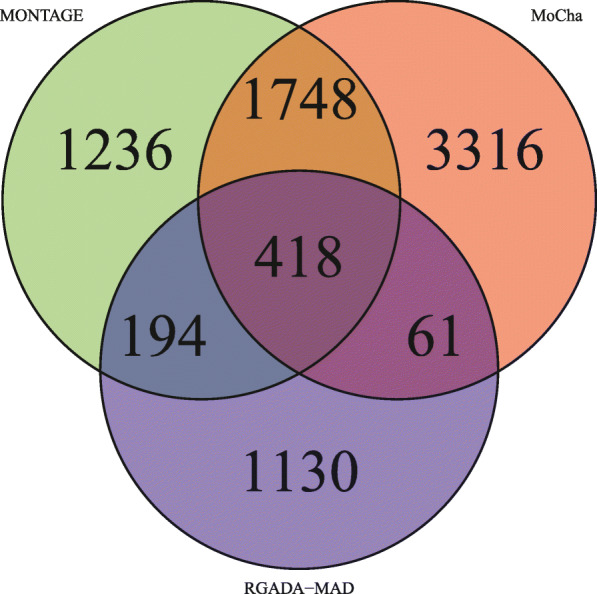
Venn Diagram Callsets Mosaic Positive Samples. We compared calling on the same sample data for 755 individuals using MONTAGE, MoCha, and RGADA-MAD. Sensitivity of 0.975 at false positive rate 0.344 (MONTAGE) vs. sensitivity of 0.920 at false positive rate 0.598 (MoCha) vs. sensitivity of 0.280 at false positive rate 0.627 (RGADA-MAD) based on 755 Illumina Human610-Quadv1 samples analyzed by all algorithms. The MONTAGE algorithm provides a competitive tool to be used for mosaic CNV detection

We compared calling on the same sample data for 755 individuals using MONTAGE, MoCha, and RGADA-MAD (Fig. 7). Using a majority voting scheme, many more mosaic CNV calls overlap MONTAGE than MoCha or RGADA-MAD. If we assume samples selected by two or more callers as true positives, then we have 1748 + 194 + 61 + 418 = 2421. Apparently MONTAGE has the highest sensitivity: 1–61/2421 = 0.975, followed by MoCha 1–194/2421 = 0.920 and RGADA-MAD 1–1748/2488 = 0.280. To compute specificity using this similar majority-vote approach, we need to know the size of the background in the background, namely, the number of samples that are considered as negatives by all three callers, which is not well defined. Alternatively, we can look at accuracy (true positive rate), which is generally a trade-off for sensitivity. Based on the Venn diagram, MoCha has the largest number of samples called by itself (3316). If we assumed the samples called by only one caller are false positives, then the accuracy (true positive rate) of MONTAGE is (1748 + 194 + 418)/ (1748 + 194 + 418 + 1236) = 0.656 vs. MoCha (1748 + 61 + 418)/(1748 + 61 + 418 + 3316) = 0.402 vs. RGADA-MAD (194 + 61 + 418)/ (194 + 61 + 418 + 1130) = 0.373. This is a better tradeoff for MONTAGE. Namely, sensitivity of 0.975 at false positive rate 0.344 (MONTAGE) vs. sensitivity of 0.920 at false positive rate 0.598 (Mocha). We note that this estimate is contingent on assumptions. While we acknowledge that these assumptions are imperfect, this analysis gives good evidence that our FDR is well-controlled. (We also note that while we cannot completely rule out the possibility that our FDR is higher than we estimated, the key results of our paper are robust to higher FDRs than estimated; e.g., we would only expect a higher-than-estimated FDR to weaken GWAS associations and decrease effect sizes.)

63% of the mosaic events identified were found in males compared to the 50% male percentage in the input dataset Fisher’s exact test (2-Tail) *p* = 1.554e-11 (Supplementary Table 2). Mosaic duplications were observed more in males and older individuals. Mosaic CNN-LOH were found to affect the sexes equally. The sample race as determined by principal components analysis is provided in Supplementary Table 3.

## Discussion

Mosaic CNVs of intermediate states between integer copy number variation are important genetic/genomic events in both clinical and research settings. However, detection of these mosaic events has been limited to incidental findings from CNV algorithms designed for integer discrete copy numbers and not the continuous nature of mosaic CNVs. For example, the tripod algorithm requires parents for calling mosaic events therefore is limited. Moreover, in a recent study using Mocha [8], 8342 mosaic chromosomal alterations (mCAs) were reported in 7585 individuals ranging in length from 50 kb–249 Mb. These mCAs were obtained from blood-derived DNA samples from 151,202 UK Biobank participants aged 40–70 years using new phase-based computational techniques (estimated false discovery rate, 6–9%). However, as 5522 of these mosaics have negative LRR values, they should be considered to be deletions. MAD was notably used in The Cancer Genome Atlas (TCGA) mosaic CNV analysis [19]. Exome sequencing (~ 8000 samples) was used to compare 22 different cancer phenotypes with more than 6000 controls using a case–control study design and demonstrate that mosaic protein truncating variants in these genes are also associated with solid-tumor cancers.

In light of shortage of high performance tools, we designed a new mosaic CNV detection tool aimed at providing high sensitivity and specificity mosaic CNV detection and fast runtime. In comparison, we show that in certain circumstances other algorithms miss critical mosaic events while overcalling other false events.

Others have shown that mosaic CNVs are enriched in males [8]. Our analysis concurs with this, showing that 63% of the mosaic events identified were found in males.

This mosaic CNV detection work has implications in cancer, cell free fetal DNA, and aging [20, 21]. Tumor-normal heterogeneity can appear similarly to germline mosaic CNV. Therefore, cancer phenotyping records are important in conditioning the assessment of supposed mosaic CNV callsets. Cell free fetal DNA is another application that such mosaic CNV detection and association presented here could be of utility. Prenatal testing could be enhanced by deconvolution of the maternal and child CNV genotype profile. Aging accumulating CNVs has been investigated previously [11, 12]. In older age cohorts which have had more exposure to potential environmental hazards inducing CNVs in subsets of cells is another important longitudinal consideration [22, 23].

## Conclusions

Mosaic CNVs represent an important class of variation in clinical genetic diagnosis that are often missed. To successfully diagnose mosaic CNVs, it’s important to develop targeted detection tools and systematically apply them to large cohorts to truly understand its relevance and frequency of mosaic CNVs in the general population. Here we demonstrate the utility of our fast scalable tool, MONTAGE, specifically designed for mosaic CNV detection. We envision MONTAGE being an integral part to include for future mosaic CNV detection and analysis.

### Availability and requirements

**-** Project name: MONTAGE.

- Project home page: https://github.com/CAG-CNV/MONTAGE

- Operating system(s): Platform independent.

- Programming language: bash, perl.

- Other requirements: NA.

- License: GNU GPL.

- Any restrictions to use by non-academics: license needed.

## Supplementary Information

**Additional file 1: Supplementary Fig. 1.** Age Distribution of Studied Cohort. **Supplementary Fig. 2.** Modeling B-allele Frequency Standard Deviation for Mosaic Copy Number States. **Supplementary Table 1.** Samples Genotyped on Illumina SNP microarray platforms. **Supplementary Table 2.** Genotyping Sex. **Supplementary Table 3.** Genotyping Race.

**Additional file 2:.**

## Data Availability

https://github.com/CAG-CNV/MONTAGE All data authorized for dbGaP submission have been deposited to dbGaP (accessions: phs000490.v1.p1, phs000607.v3.p2, phs000371.v1.p1, phs000490.v1.p1, phs001194.v2.p2, phs001194.v2.p2.c1, phs001661, and phs000233).

## Abbreviations

CNV: Copy number variation
BAF: B-allele frequency
LRR: Log R ratio
MAD: Mosaic alteration detection
MCA: Mosaic chromosomal alteration
