# Supplementary material for: MONTAGE: a new tool for high-throughput detection of mosaic copy number variation
Source: BMC Genomics. 2021 Feb 24;22:133. doi: 10.1186/s12864-021-07395-7 (PMC7905641; doi:10.1186/s12864-021-07395-7)
Supplement: Supplementary file 1 — Additional file 1: Supplementary Fig. 1. Age Distribution of Studied Cohort. Supplementary Fig. 2. Modeling B-allele Frequency Standard Deviation for Mosaic Copy Number States. Supplementary Table 1. Samples Genotyped on Illumina SNP microarray platforms. Supplementary Table 2. Genotyping Sex. Supplementary Table 3. Genotyping Race. [file 12864_2021_7395_MOESM1_ESM.docx]

**Additional files**

- File name: Additional file 1

- Title of data: Samples Genotyped on Illumina SNP microarray platforms

- Description of data: Illumina SNP microarray versions used in this study to validate consistency of mosaicism detection.

**Supplement**

Supplementary Figure 1. Age Distribution of Studied Cohort


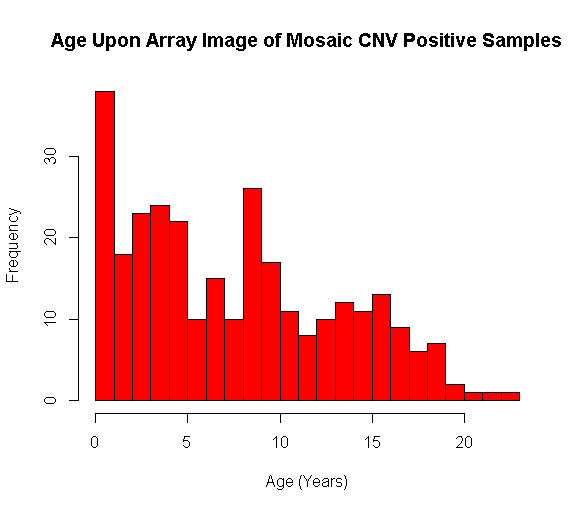


Supplementary Figure 2. Modeling B-allele Frequency Standard Deviation for Mosaic Copy Number States

**
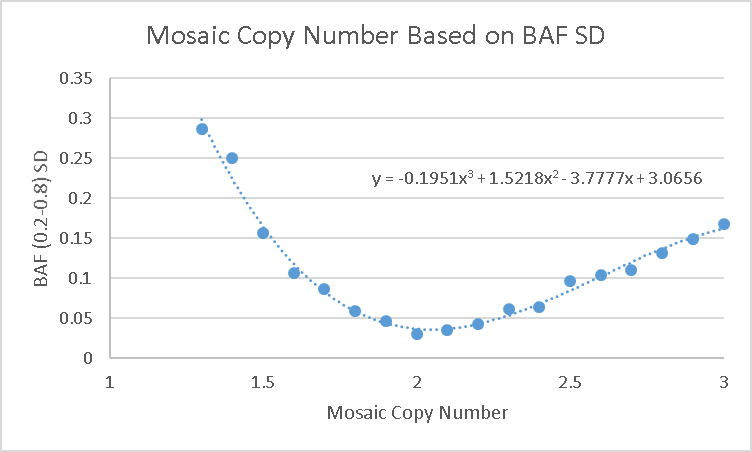
**

**Supplementary Table 1.** Samples Genotyped on Illumina SNP microarray platforms.

| Count | IlluminaProduct/ChipVersion |
| --- | --- |
| 60666 | GSAMD-24v1-0 |
| 43047 | Human610-Quadv1 |
| 31594 | CVDSNP55 |
| 18852 | HumanExome-12v1-1 |
| 18321 | HumanHap550v3 |
| 17759 | HumanOmniExpress-12v1 |
| 17231 | HumanHap550-2_v3-1 |
| 14592 | UNIVERSITY-HOMOSAPIEN-81 |
| 13650 | GSAMD-24v2-0 |
| 12354 | BDCHP-1X10-HUMANHAP550 |
| 11946 | GSA-mGluR_enrichd |
| 8999 | iSelect 86k |
| 8810 | Omni2-5-8v1-3 |
| 8656 | Human660W-Quad_v1 |
| 8024 | HumanOmniExpressExome-8v1-2 |
| 7812 | IBD_Consort_ExomePlus |
| 7044 | HumanExome-12v1 |
| 6907 | Human1M-Duov3-0 |
| 6809 | CVD_ _49234 |
| 6356 | GSA |
| 6223 | HumanOmni1-Quadv1-0 |
| 4959 | CytoSNP-850K_v1-1 |
| 4767 | HumanOmniExpressExome-8v1 |
| 4606 | HumanOmniExpress-12v1-1 |
| 4423 | CytoSNP-850K |
| 4296 | Cardio-Metabo_Chip |
| 3984 | HumanOmniExpress-24v1-0 |
| 3923 | MEGA_Consortium_v2 |
| 3080 | HumanOmni25-8v1 |
| 2952 | iSelect_CVD3 |
| 2640 | HumanOmniExpress-24v1-1 |
| 2568 | iSelect 86k |
| 1968 | 610Q |
| 1824 | HumanCoreExome-12v1-0 |
| 1536 | 610Q |
| 1366 | HumanCore-24v1-0 |
| 1344 | 610Q |
| 1320 | HumanOmni25-8v1-1 |
| 1288 | CytoSNP-850Kv1-2 |
| 1152 | Exomev1-1-Neurov1-0-NeuroX |
| 1032 | Immuno_BeadChip |
| 996 | Multi-EthnicGlobal |
| 982 | HumanOmni25-4v1 |
| 960 | OmniExpressExome-8v1-4 |
| 872 | HumanOmni25-8v1-2 |
| 852 | HumanCore-12v1-0 |
| 672 | Cyto |
| 608 | HumanOmni25Exome-8v1-2 |
| 600 | HumanCoreExome-24v1-0 |
| 226 | HumanExon510S-2\ v1.0 |
| 192 | PsychArray |
| 192 | HumanOmniExpressExome-8v1-3 |
| 152 | Omni2-5-8v1-4 |
| 93 | HumanCytoSNP-12v1-0 |
| 77 | HumanOmni25-4v1MultiUse |
| 48 | HumanCytoSNP-12v2 |
| 48 | HumanCNV-12_v1-0 |
| 40 | OmniExpressExome-8v1-6 |
| 16 | Core-24v1-2 |

Supplementary Table 2. Genotyping Sex

| Genotyping Sex (Plink --check-sex) | Count |
| --- | --- |
| M | 162,189 |
| F | 135,370 |
| U | 11,021 |

Supplementary Table 3. Genotyping Race

| Genotyping Race (Plink --pca) | COUNT |
| --- | --- |
| EUROPEAN | 74,133 |
| AFRICAN | 14,057 |
| UNASSIGNED | 8,182 |
| MEXICAN | 2,816 |
| EAST ASIAN | 1,140 |
